# Fracture toughness of three-dimensional printed and milled denture bases

**DOI:** 10.1371/journal.pone.0329556

**Published:** 2025-08-25

**Authors:** Walaa A. Babeer

**Affiliations:** Oral and Maxillofacial Prosthodontics, King Abdulaziz University, Jeddah, Saudi Arabia; International Medical University, MALAYSIA

## Abstract

Denture base fractures are a common issue, particularly in overdentures, necessitating high fracture toughness in denture base polymers to withstand oral mechanical loads. Despite the importance of fracture resistance, current denture base materials often fall short of meeting the required standards. The literature shows conflicting results regarding the fracture toughness of three-dimensional (3D)-printed materials compared to conventional and milled options. This study aimed to address this gap by evaluating the fracture toughness of commercially available digital denture base resins, including two CAD/CAM-milled and two 3D-printed materials. Using a three-point bending test on notched beams according to ISO 20795–1:2013 standards, the study compared the fracture toughness of FormLabs denture base LP, NextDent Denture 3D, AvaDent, and XANTECH PMMA. Results revealed that NextDent exhibited the highest fracture toughness, suggesting better crack propagation resistance. Printed acrylic (NextDent) and prepolymerized polymethyl methacrylate (AvaDent and XANTECH) demonstrated superior fracture toughness compared to Formlabs photopolymerized diurethane methacrylate. However, none of the tested materials met the ISO’s highest impact resistance requirements. These findings highlight the variability in fracture toughness among different manufacturing techniques and compositions, emphasizing the need for further research to develop denture base materials with enhanced impact resistance. This study provides valuable insights for clinicians in material selection for denture fabrication and underscores the importance of continued innovation in dental materials to improve the longevity and performance of prosthetic devices.

## Introduction

The edentulous population is growing worldwide owing to increased life expectancy, resulting in a greater need for removable dentures [1]. Polymethyl methacrylate (PMMA) acrylic remains the standard denture base material because many patients cannot afford implant-supported or ceramic alternatives [2]. PMMA offers favorable aesthetics and ease of processing, but its inherent fracture resistance is poor [3]. This limitation has led to frequent denture fractures, sparking efforts to reinforce or modify the polymer for greater strength [4]. Nevertheless, fractures of PMMA bases remain a common complication—particularly in implant overdentures that have higher rates of base or tooth fractures compared to other prothesis types [5,6]. These clinical challenges underscore the need for denture base materials with superior strength and durability. To address the problem of denture fractures, it is crucial to employ mechanical tests that adequately reflect clinical conditions.

Several standardized methods are available to evaluate polymer fracture resistance, including notched Izod and Charpy impact tests and a modified three-point bending test for fracture toughness [7]. Comparative studies have shown that these different methods can yield markedly different results for the same PMMA material, making it difficult to determine which test best predicts clinical performance [7]. Impact tests like Charpy measure the energy required to break a notched specimen under a sudden blow, analogous to dropping a denture [7,8]. The resulting impact strength is influenced by specimen geometry and test configuration [7]. By contrast, fracture toughness testing measures a material's resistance to slow crack propagation [9] and often more sensitively reflects the effect of toughening modifications in the resin [7]. Fracture toughness is particularly relevant for denture polymers, since cracks can initiate and grow under repeated masticatory forces or impacts, leading to failure [10]. Accordingly, standards for denture base polymers (ISO 20795−1) include fracture toughness criteria in their testing protocols [11]. In general, a polymer’s fracture toughness is regarded as one of its most critical mechanical properties [12]. It serves as a reliable indicator of structural performance across different dental materials including ceramics, denture base resins, composite restoratives, and even metallic alloys [12–14] and has been used to characterize the strength of acrylic denture base materials [13].

In parallel with material improvements, denture fabrication techniques have advanced through digital manufacturing. Two primary digital workflows are computer-aided milling (subtractive manufacturing) and three-dimensional (3D) printing (additive manufacturing) [15]. Milled dentures are precision-carved from pre-polymerized PMMA discs, producing a dense acrylic base; for example, the AvaDent system uses highly cross-linked PMMA blocks that integrate denture teeth into the base during milling [15]. Digitally fabricated dentures offer several clinical advantages over conventional flask-and-pack acrylics, including improved fit and a faster, more cost-effective production process [16–18]. 3D-printed denture (additive) bases are built layer by layer from liquid resin, and their mechanical properties can vary with the printing technology (e.g., stereolithography vs. digital light processing) and post-curing process. Milled bases also generally achieve superior adaptation compared to 3D-printed or conventionally processed bases, although studies report that SLA-printed dentures can attain a similarly high level of fit accuracy [19]. Additionally, the low porosity of pre-polymerized milled PMMA may improve hygiene by reducing microbial accumulation. For example, *Candida albicans* has been shown to adhere less to milled denture surfaces than to conventional acrylic surfaces [20,21].

Despite these benefits, it remains uncertain how the newer milled and printed denture base materials compare in terms of fracture resistance under functional loads. Denture base fractures persist as a clinical concern, highlighting the need for improved material toughness. Given the limited and sometimes conflicting data on the fracture toughness of milled versus 3D-printed denture bases, a direct comparison of these digital fabrication methods is needed. Therefore, the present study aimed to evaluate the fracture toughness of representative denture base materials fabricated using modern digital techniques (milling and 3D printing). Fracture toughness testing was conducted in accordance with ISO 20795−1 guidelines for denture base polymers [11]. The hypothesis was that there would be no significant difference in fracture toughness between the milled and printed denture base resins.

## Materials and methods

### Specimen grouping and material information

A total of 40 rectangular beam specimens were prepared, with 10 specimens (n = 10) per group. Four different commercially available denture base materials were used, representing both milled and 3D-printed manufacturing techniques. The materials, compositions, and preparation methods are summarized in Table 1. Ethics approval was not required for this in vitro study.

**Table 1:** Material type, brand, compositions, and processing method.

Material	Brand name	Composition	Preparation and Polymerization
**Prepolymerized milled PMMA blocks**	AvaDent (AvaDent Digital Dental Solutions, Scottsdale, AZ, USA)	Prepolymerized PMMA (PMMA 99.5%, pigments < 1.0%)	Discs were cut to the required dimension with the milling machine Ceramill Motion 2, and the Amann Girrbach (AG).
**Prepolymerized milled PMMA blocks Chinese brand**	XANTECH PMMA (XANTECH PMMA; Nanyang Liandong Biotechnology Co. Ltd, Henan, China)	Prepolymerized PMMA (PMMA>99%; Toner<1%	Discs were cut to the required dimension with the milling machine Ceramill Motion 2, and the Amann Girrbach (AG).
**3d printed Acrylic aster**	NextDent Denture 3D (NextDent, Vertex-Dental B.V, Soesterberg, The Netherlands)	Ethoxylated bisphenol A dimethacrylate 7,7,9 (or 7,9,9)-trimethyl-4,13-dioxo-3,14-dioxa-5,12- diazahexadecane-1,16-diyl bismethacrylate 2- hydroxyethyl methacrylate. silicon dioxide diphenyl (2,4,6- trimethylbenzoyl) phosphine oxide titanium dioxide	Printer: NextDent 5100 (Vertex-Dental B.V, Soesterberg, The Netherlands) Orientation: 90° Layer thickness: 50 μm Cleaning: in >90% ethanol for 5 minutes total cleaning time. Post curing time: 30 minutes/60°C
**3d printed diurenthane methacrylate**	FormLabs denture base LP (FormLabs Inc, Somerville, MA, USA)	55–75% w/w urethane dimethacrylate, 15–25% w/w methacrylate monomers, and < 0.9% w/w phenyl bis (2,4,6-trimethylbenzoyl)-phosphine oxide	Printer: FormLabs 3B (FormLabs Inc, Somerville, MA, USA) Orientation: 97° Layer thickness: 50 μm Cleaning: Isopropyl alcohol 97%, Saudi Pharmaceutical Industries, Riyadh, KSA) Post curing: No need for post curing according to the manufacturer’s instructions

### Specimen design and fabrication

**Fig 1:**
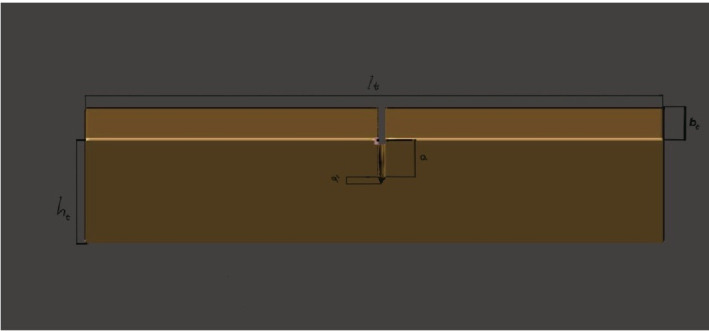
Fracture toughness sample CAD design.

All specimens were designed in STL (Standard Tessellation Language) format based on the specifications of ISO 20795–1:2013, with target dimensions of 8.0 ± 0.2 mm in height, 4.0 ± 0.2 mm in width, and a span length of 32.0 ± 0.1 mm. A pre-crack of 3.0 ± 0.2 mm depth was digitally incorporated into each CAD model to ensure consistency (Fig 1). The final notch was manually extended using a hand-held razor blade to initiate crack propagation. Glycerin was applied during the notch process to minimize friction, and the notch depth was verified under a light microscope. All specimens were visually inspected to confirm uniformity of the final crack geometry.

### Manufacturing techniques

#### Milled groups.

**Fig 2:**
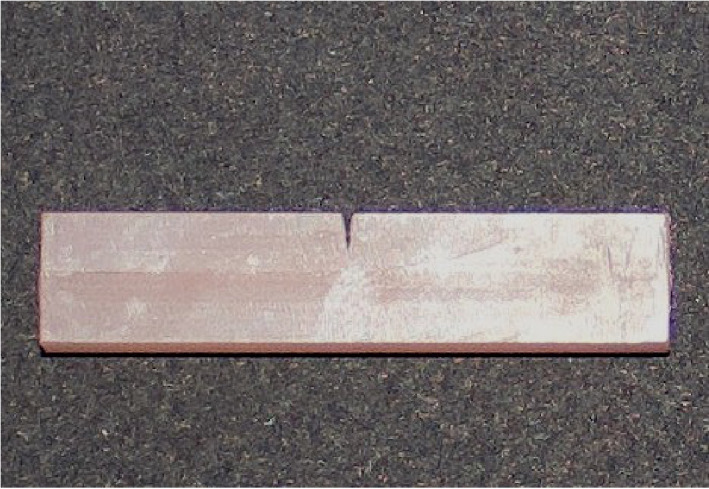
Milled AvaDent sample.

Specimens from AvaDent and Xantech PMMA discs were CAD designed (Fig 1) using Meshmixer and milled with a Ceramill Motion 2 milling machine (Amann Girrbach, Germany). These pre-polymerized PMMA blocks were processed according to manufacturer recommendations (Fig 2).

#### 3D-printed groups.

Using the same CAD design, Formlabs and NextDent specimens were fabricated using SLA (Form 3B, Formlabs Inc., USA) and DLP (NextDent 5100, Vertex Dental, Netherlands) technologies, respectively. Layer thickness was set at 50 μm for both. STL files were oriented at 90° (NextDent) and 97° (Formlabs) for optimal mechanical properties. Supports were removed post-printing. Formlabs specimens were not post-cured as per manufacturer guidelines, while NextDent samples were post-cured for 30 minutes at 60°C in a NextDent LC-3DPrint Box.

**Fig 3:**
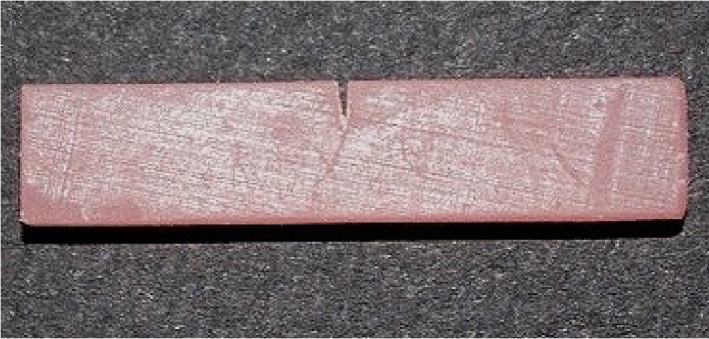
Printed Formlabs sample.

Post-processing steps, including ethanol or IPA washing, were performed as per each manufacturer’s instructions. The common procedures of support removal and cleaning were standardized across groups and performed by a single trained operator (Fig 3).

### Conditioning and test environment

All specimens were stored in distilled water at 37°C for 7 days to simulate oral conditions. Prior to testing, specimens were equilibrated at 23°C for 1 hour. Testing was conducted under ambient laboratory humidity (approximately 50%) without additional humidity control.

### Fracture toughness testing

**Fig 4:**
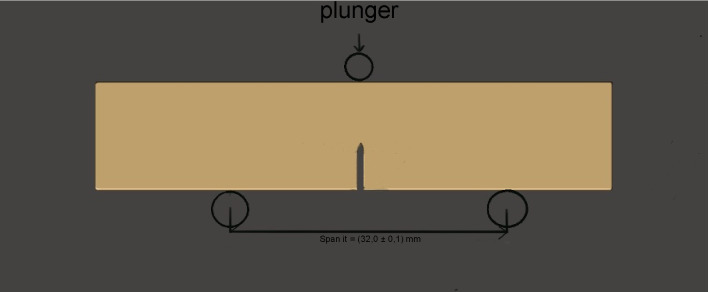
Location of force application (F) in test setup.

Mechanical testing followed ISO 20795–1:2013 using a three-point bending configuration. Each beam was loaded at a crosshead speed of 1.0 ± 0.2 mm/min with a 32 mm support span (Fig 4).

Fracture toughness (Kmax) was calculated using the following formula:


$$\begin{array}{c} Kmax=\frac{fP_{max}l_{t}}{b_{t}{h_{t}}^{3/2}}X\surd 10^{-3}MPa\times m^{1/2} \end{array}$$

where

ƒ is a geometrical function dependent on x,


$$f\left(x\right)=3x^{1/2}[1,99-x\left(1-x\right)\left(2,15-3,93x+2,7x^{2}\right]/[2\left(1+2x\right){\left(1-x\right)}^{3/2}],$$

$$x\mathrm{\backslash nolimits}=a/h_{t},$$

P_max_ is the maximum load exerted on the specimen in Newtons, and a_t_, h_t_, w_t_, and l_t_ are expressed in millimeters (Fig 1).

### Experimental design and bias control

Specimens were assigned to groups based on the denture base material and manufacturing technique. The operator performing the testing was blinded to the material group to reduce experimental bias. Each measurement was recorded by an assistant not involved in sample fabrication or data analysis.

### Statistical analysis

Normality was evaluated using the Kolmogorov–Smirnov and Shapiro–Wilk tests, confirming that the data were normally distributed. One-way analysis of variance (ANOVA) was performed to determine whether significant differences existed among the four material groups. When ANOVA revealed significance, Bonferroni post hoc tests were conducted to assess pairwise differences between groups. The level of significance was set at α = 0.05. Effect size was calculated using eta squared (η^2^). All analyses were performed using IBM SPSS Statistics (Version 23.0; IBM Corp., Armonk, NY, USA).

### Post-hoc power analysis and sample justification

A post-hoc power analysis was performed based on the observed effect size from the one-way ANOVA (η^2^ = 0.769). Given the total sample size of 40 (n = 10 per group), the power of the test exceeded 0.99 at an alpha level of 0.05. This indicates that the sample size was sufficient to detect statistically significant differences among the groups with a very high probability, minimizing the risk of Type II error. The large effect size and highly significant ANOVA results (F(3,36) = 39.902, p < 0.001) support the robustness of the findings.

## Results

**Fig 5:**
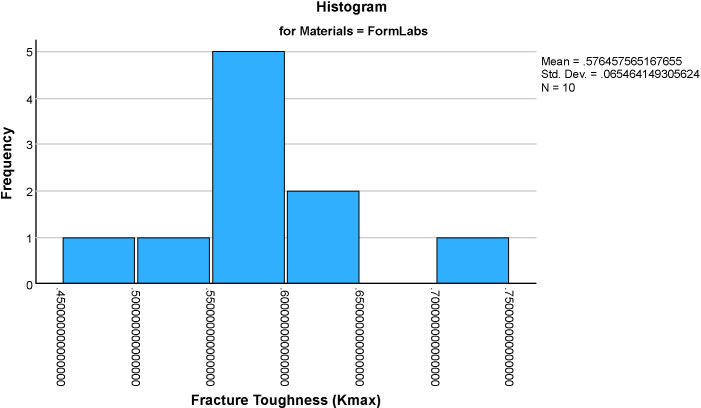
Histograms showing the distribution of fracture toughness (Kmax) values for four dental materials (Formlabs). Each histogram illustrates the frequency and spread of Kmax within each material group, aiding in the assessment of data normality and variability.

**Fig 6:**
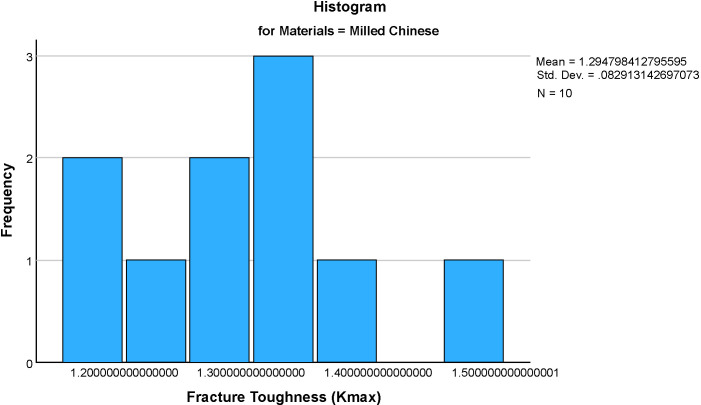
Histograms showing the distribution of fracture toughness (Kmax) values for four dental materials (Milled Chinese). Each histogram illustrates the frequency and spread of Kmax within each material group, aiding in the assessment of data normality and variability.

**Fig 7:**
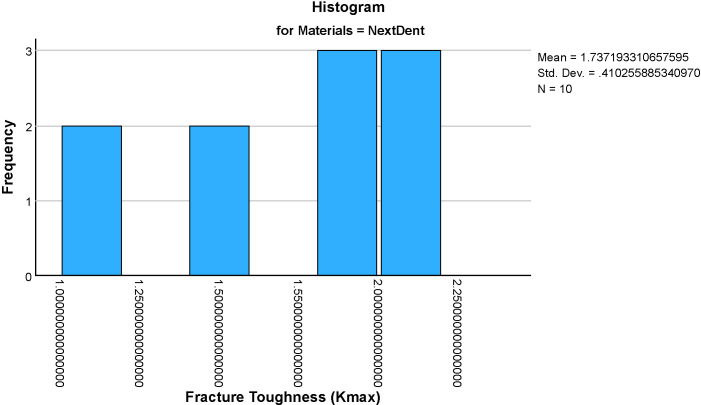
Histograms showing the distribution of fracture toughness (Kmax) values for four dental materials (NextDent). Each histogram illustrates the frequency and spread of Kmax within each material group, aiding in the assessment of data normality and variability.

**Fig 8:**
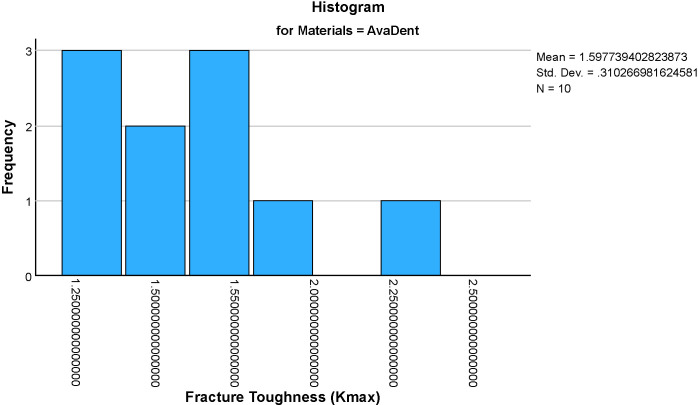
Histograms showing the distribution of fracture toughness (Kmax) values for four dental material (AvaDent). Each histogram illustrates the frequency and spread of Kmax within each material group, aiding in the assessment of data normality and variability.

**Fig 9:**
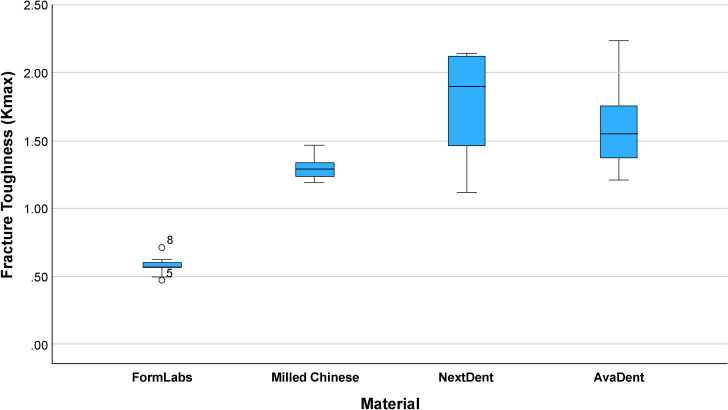
Boxplot showing the distribution of fracture toughness (Kmax) across four dental materials.

The mean fracture toughness (Kmax)values for the four groups were: Formlabs: 0.576 ± 0.065 MPa·m^1/2^, Milled Chinese: 1.295 ± , MPa·m^1/2^, NextDent: 1.737 ± 0.401 MPa·m^1/2^ and AvaDent: 1.598 ± 0.310 MPa·m^1/2^ (Figs 5–9). Descriptive statistics of the tested materials are summarized in Table 2.

**Table 2:** Descriptive statistics: mean, standard deviation, 95% confidence interval, and range of tested materials.

Descriptives								
Fracture Toughness (Kmax)								
Materials	N	Mean	Std. Deviation	Std. Error	95% Confidence Interval for Mean		Minimum	Maximum
					Lower Bound	Upper Bound		
**Formlabs**	10	.576457565167655	.065464149305623	.020701581689110	.529627333868383	.623287796466927	.472073826659547	.711168976603683
**Milled Chinese**	10	1.294798412795595	.082913142697073	.026219437888531	1.235485923571513	1.354110902019677	1.191494965827777	1.461536672298191
**NextDent**	10	1.737193310657595	.401255885340970	.126888252222483	1.450152142017559	2.024234479297632	1.115214794205744	2.141087645624427
**AvaDent**	10	1.597739402823873	.310266981624581	.098115034467928	1.375787774824056	1.819691030823690	1.207839756020426	2.231100957470412
**Total**	40	1.301547172861180	.517614524909605	.081842042435016	1.136006016750633	1.467088328971726	.472073826659547	2.231100957470412

One-way ANOVA showed a statistically significant difference between the groups (F(3, 36) = 39.902, P < 0.001), with a large effect size (η^2^ = 0.769).

### Post-hoc comparisons

Bonferroni post hoc tests indicated: Formlabs had significantly lower (Kmax) than all other groups (P < 0.001). NextDent was significantly higher than Milled Chinese (P = 0.003), but not significantly different from AvaDent (P = 1.000). Milled Chinese and AvaDent were not significantly different from each other (P = 0.078). These results were consistent with Tukey’s HSD test (Table 3).

**Table 3:** Post-hoc test, multiple comparisons, and significant values.

Multiple Comparisons							
Dependent Variable: Fracture Toughness (Kmax)							
	(I) Material	(J) Material	Mean Difference (I-J)	Std. Error	Sig.	95% Confidence Interval	
						Lower Bound	Upper Bound
Tukey HSD	Formlabs	Milled Chinese	−.718340847627940^*^	.115851635612841	<.001	−1.030355619605818	−.406326075650062
		NextDent	−1.160735745489940^*^	.115851635612841	<.001	−1.472750517467818	−.848720973512062
		AvaDent	−1.021281837656218^*^	.115851635612841	<.001	−1.333296609634096	−.709267065678340
	Milled Chinese	Formlabs	.718340847627940^*^	.115851635612841	<.001	.406326075650062	1.030355619605818
		NextDent	−.442394897862001^*^	.115851635612841	.003	−.754409669839878	−.130380125884123
		Avadent	−.302940990028278	.115851635612841	.060	−.614955762006156	.009073781949600
	NextDent	Formlabs	1.160735745489940^*^	.115851635612841	<.001	.848720973512062	1.472750517467818
		Milled Chinese	.442394897862001^*^	.115851635612841	.003	.130380125884123	.754409669839878
		Avadent	.139453907833722	.115851635612841	.629	−.172560864144156	.451468679811600
	Avadent	Formlabs	1.021281837656218^*^	.115851635612841	<.001	.709267065678340	1.333296609634096
		Milled Chinese	.302940990028278	.115851635612841	.060	−.009073781949600	.614955762006156
		NextDent	−.139453907833722	.115851635612841	.629	−.451468679811600	.172560864144156
Bonferroni	Formlabs	Milled Chinese	−.718340847627940^*^	.115851635612841	<.001	−1.041795363739077	−.394886331516803
		NextDent	−1.160735745489940^*^	.115851635612841	<.001	−1.484190261601077	−.837281229378803
		Avadent	−1.021281837656218^*^	.115851635612841	<.001	−1.344736353767355	−.697827321545081
	Milled Chinese	Formlabs	.718340847627940^*^	.115851635612841	<.001	.394886331516803	1.041795363739077
		NextDent	−.442394897862001^*^	.115851635612841	.003	−.765849413973137	−.118940381750864
		Avadent	−.302940990028278	.115851635612841	.078	−.626395506139415	.020513526082859
	NextDent	Formlabs	1.160735745489940^*^	.115851635612841	<.001	.837281229378803	1.484190261601077
		Milled Chinese	.442394897862001^*^	.115851635612841	.003	.118940381750864	.765849413973137
		Avadent	.139453907833722	.115851635612841	1.000	−.184000608277415	.462908423944859
	Avadent	Formlabs	1.021281837656218^*^	.115851635612841	<.001	.697827321545081	1.344736353767355
		Milled Chinese	.302940990028278	.115851635612841	.078	−.020513526082859	.626395506139415
		NextDent	−.139453907833722	.115851635612841	1.000	−.462908423944859	.184000608277415

*The mean difference is significant at the 0.05 level.

### Homogeneous subsets

According to Tukey’s homogeneous subsets Table 4, Formlabs was isolated as the lowest-performing group. Milled Chinese and AvaDent formed an intermediate group. NextDent was the top-performing group, significantly distinct from Formlabs and Milled Chinese but not from AvaDent.

**Table 4:** Homogeneous subsets.

Fracture Toughness (Kmax)					
	Material	N	Subset for alpha = 0.05		
			1	2	3
**Tukey HSD** ^**a**^	Formslabs	10	.576457565167655		
	Milled Chinese	10		1.294798412795595	
	Avadent	10		1.597739402823873	1.597739402823873
	NextDent	10			1.737193310657595
	Sig.		1.000	.060	.629

Means for groups in homogeneous subsets are displayed.

a. Uses harmonic mean of sample sizes = 10.000.

## Discussion

The maximum intensity factor (KIc) differed significantly among the groups, thereby rejecting the null hypothesis (H01). Furthermore, none of the materials tested met the ISO 20795–1:2013 criteria for high-impact resistance, which requires at least eight out of ten specimens to exhibit a fracture toughness of not less than 1.9 MPa·m^1/2^. Specifically, Formlabs demonstrated the lowest mean KIc (.576 ± 0.0655 MPa·m^1/2^), clearly below the ISO threshold. This result aligns with previous research, which reported Formlabs photopolymerized diurethane methacrylate to have a significantly lower fracture toughness compared to CAD/CAM-milled materials [22]. Similarly, the inferior fracture toughness of Formlabs relative to prepolymerized polymethyl methacrylate (PMMA), such as AvaDent, was also consistent with earlier findings [23].

Formlabs’ Fracture toughness was substantially lower than that of NextDent, possibly owing to notable differences in monomer composition and manufacturing techniques [24]. NextDent employs digital light processing (DLP) technology, whereas Formlabs uses stereolithography (SLA). The DLP approach utilizes a projector-based curing process, which differs fundamentally from the laser-based curing method employed by SLA [25]. Additionally, NextDent’s resin consists primarily of acrylic esters and ethoxylated bisphenol A dimethacrylate, while Formlabs uses urethane dimethacrylate [26]. The structural variances in these monomers lead to distinctly different polymer network architectures and, consequently, their physical and mechanical properties [27]. Previous studies highlight that variations among different 3D-printed denture materials significantly affect their mechanical behavior [28]. The fracture toughness and flexural strength of CAD/CAM denture bases, printed or milled, critically depend on their composition [29].

An important factor influencing the mechanical properties of 3D-printed denture bases is the duration and methodology of post-curing. Optimal curing durations significantly enhance the properties, typically observed up to 20 minutes [30]. In this study, NextDent was post-cured for 30 minutes following manufacturer guidelines, while Formlabs received no additional curing. This disparity in curing protocols may partially explain the superior fracture toughness performance of NextDent.

Further influencing properties, build parameters such as layer thickness and orientation, as well as post-processing methods like support removal and washing, considerably affect the mechanical outcomes of printed denture bases [31,32]. Typically, printed materials show superior resistance to crack propagation compared to milled bases, likely attributed to their unique polymer network and propagation behavior involving material crazing or microcracking around propagating cracks until reaching an arrest point [33]. NextDent demonstrated the highest KIc among all tested groups, supporting this general trend and previous research findings [34].

Milled denture bases generally exhibit comparable or superior mechanical properties relative to conventional heat-polymerized resins [24,35]. AvaDent, specifically, has been reported to have greater flexural strength and hardness than traditional heat-polymerized denture bases [36,37]. Notably, previous investigations reported lower fracture toughness values for Formlabs compared to AvaDent, supporting the current results [38].

This study has several limitations. Although a post-hoc power analysis indicated that the sample size (n = 10 per group) was sufficient to detect the observed large effect size (η^2^ = 0.769) with high statistical power (>0.99), the generalizability of the findings remains constrained. The materials were tested in a controlled in vitro setting, with fracture toughness measured after 7 days of water storage at 37°C, but without simulation of long-term clinical conditions, such as thermal cycling, salivary exposure, or repeated mechanical loading. These environmental and functional variables may affect real-world material performance. Additionally, only four denture base materials were examined, which limits the extrapolation of results to other brands, formulations, or manufacturing parameters not included in this study. Moreover, each material group was fabricated using a single production batch, which ensured internal consistency but may not fully capture the impact of inter-batch variability. While the findings demonstrate robust internal validity, caution is warranted when extending conclusions to broader clinical applications. Future research should incorporate more diverse material types, extended aging protocols, and in vivo or clinically simulated settings to enhance external validity.

Despite none of the tested materials meeting ISO’s high-impact resistance requirements, the clinical implications of these findings are significant. Clinicians should carefully select materials based on their specific mechanical needs. Materials demonstrating lower fracture toughness, such as Formlabs, may require caution or additional reinforcement in clinical settings prone to higher mechanical stresses. Further research exploring enhanced impact-resistant materials and the clinical relevance of fracture toughness measurements in dental practice is warranted.

In summary, the fracture toughness of digital denture base materials varies significantly depending on their composition and manufacturing process, with NextDent demonstrating superior performance compared to AvaDent, XANTECH, and Formlabs. Although none of the evaluated materials met ISO criteria, these results highlight critical considerations for future material development and clinical selection criteria.

## Conclusion

Within the limitations of this study, NextDent exhibited superior fracture toughness among tested materials, followed by AvaDent, Milled Chinese PMMA, and Formlabs. However, none of the evaluated materials met the ISO criteria for high-impact denture base classification. Clinicians should carefully consider fracture toughness properties when selecting materials to minimize prosthetic failures. Further research should focus on improving material toughness and assessing long-term clinical performance.

## Supporting information

S1 File Raw Data Spreadsheet. (XLSX)

S2 File ANOVA results. (DOCX)

S3 File Post hoc power analysis. (DOCX)
