# Supplementary material for: Fracture toughness of three-dimensional printed and milled denture bases
Source: PLoS One. 2025 Aug 25;20(8):e0329556. doi: 10.1371/journal.pone.0329556 (PMC12377610; doi:10.1371/journal.pone.0329556)
Supplement: S2 File — (DOCX) [file pone.0329556.s002.docx]

Supplemental Information 2

**Statistical analysis**

Regarding the ANOVA assumptions:

The data are not distributed normally because the Shapiro–Wilk test was not significant.

The homogeneity of the variance is significant.

There are a few outliers, but they are within range—no need for outlier removal.

Results:

Table 2 presents the means, standard deviations of the tested materials, the 95% confidence interval of the mean, and the minimum and maximum values.

| Descriptives | | | | | | | | |  |
| --- | --- | --- | --- | --- | --- | --- | --- | --- | --- |
| Fracture Toughness (Kmax) | | | | | | | | |  |
|  | **N** | **Mean** | **Std. Deviation** | **Std. Error** | **95% Confidence Interval for Mean** | | **Minimum** | **Maximum** | |
|  |  |  |  |  | **Lower Bound** | **Upper Bound** |  |  |  |
| Formlabs | 10 | .576457565167655 | .065464149305623 | .020701581689110 | .529627333868383 | .623287796466927 | .472073826659547 | .711168976603683 | |
| Milled Chinese | 10 | 1.294798412795595 | .082913142697073 | .026219437888531 | 1.235485923571513 | 1.354110902019677 | 1.191494965827777 | 1.461536672298191 | |
| NextDent | 10 | 1.737193310657595 | .401255885340970 | .126888252222483 | 1.450152142017559 | 2.024234479297632 | 1.115214794205744 | 2.141087645624427 | |
| AvaDent | 10 | 1.597739402823873 | .310266981624581 | .098115034467928 | 1.375787774824056 | 1.819691030823690 | 1.207839756020426 | 2.231100957470412 | |
| Total | 40 | 1.301547172861180 | .517614524909605 | .081842042435016 | 1.136006016750633 | 1.467088328971726 | .472073826659547 | 2.231100957470412 | |

The ANOVA is (F(3, 36) = 39.902, P < 0.001)

The effect size, represented by eta squared, is .769, indicating a large effect.

Table 3 presents the post-hoc test, multiple comparisons, and significant values.

| Multiple Comparisons | | | | | | | |
| --- | --- | --- | --- | --- | --- | --- | --- |
| Dependent Variable: Fracture Toughness (Kmax) | | | | | | | |
|  | **(I) Material** | **(J) Material** | **Mean Difference (I-J)** | **Std. Error** | **Sig.** | **95% Confidence Interval** | |
|  |  |  |  |  |  | **Lower Bound** | **Upper Bound** |
| Tukey HSD | Formlabs | Milled Chinese | -.718340847627940^*^ | .115851635612841 | <.001 | -1.030355619605818 | -.406326075650062 |
|  |  | NextDent | -1.160735745489940^*^ | .115851635612841 | <.001 | -1.472750517467818 | -.848720973512062 |
|  |  | AvaDent | -1.021281837656218^*^ | .115851635612841 | <.001 | -1.333296609634096 | -.709267065678340 |
|  | Milled Chinese | Formlabs | .718340847627940^*^ | .115851635612841 | <.001 | .406326075650062 | 1.030355619605818 |
|  |  | NextDent | -.442394897862001^*^ | .115851635612841 | .003 | -.754409669839878 | -.130380125884123 |
|  |  | Avadent | -.302940990028278 | .115851635612841 | .060 | -.614955762006156 | .009073781949600 |
|  | NextDent | Formlabs | 1.160735745489940^*^ | .115851635612841 | <.001 | .848720973512062 | 1.472750517467818 |
|  |  | Milled Chinese | .442394897862001^*^ | .115851635612841 | .003 | .130380125884123 | .754409669839878 |
|  |  | Avadent | .139453907833722 | .115851635612841 | .629 | -.172560864144156 | .451468679811600 |
|  | Avadent | Formlabs | 1.021281837656218^*^ | .115851635612841 | <.001 | .709267065678340 | 1.333296609634096 |
|  |  | Milled Chinese | .302940990028278 | .115851635612841 | .060 | -.009073781949600 | .614955762006156 |
|  |  | NextDent | -.139453907833722 | .115851635612841 | .629 | -.451468679811600 | .172560864144156 |
| Bonferroni | Formlabs | Milled Chinese | -.718340847627940^*^ | .115851635612841 | <.001 | -1.041795363739077 | -.394886331516803 |
|  |  | NextDent | -1.160735745489940^*^ | .115851635612841 | <.001 | -1.484190261601077 | -.837281229378803 |
|  |  | Avadent | -1.021281837656218^*^ | .115851635612841 | <.001 | -1.344736353767355 | -.697827321545081 |
|  | Milled Chinese | Formlabs | .718340847627940^*^ | .115851635612841 | <.001 | .394886331516803 | 1.041795363739077 |
|  |  | NextDent | -.442394897862001^*^ | .115851635612841 | .003 | -.765849413973137 | -.118940381750864 |
|  |  | Avadent | -.302940990028278 | .115851635612841 | .078 | -.626395506139415 | .020513526082859 |
|  | NextDent | Formlabs | 1.160735745489940^*^ | .115851635612841 | <.001 | .837281229378803 | 1.484190261601077 |
|  |  | Milled Chinese | .442394897862001^*^ | .115851635612841 | .003 | .118940381750864 | .765849413973137 |
|  |  | Avadent | .139453907833722 | .115851635612841 | 1.000 | -.184000608277415 | .462908423944859 |
|  | Avadent | Formlabs | 1.021281837656218^*^ | .115851635612841 | <.001 | .697827321545081 | 1.344736353767355 |
|  |  | Milled Chinese | .302940990028278 | .115851635612841 | .078 | -.020513526082859 | .626395506139415 |
|  |  | NextDent | -.139453907833722 | .115851635612841 | 1.000 | -.462908423944859 | .184000608277415 |
| *. The mean difference is significant at the 0.05 level. | | | | | | | |

Table 4 Homogeneous subsets

| **Fracture Toughness (Kmax)** | | | | | |
| --- | --- | --- | --- | --- | --- |
|  | Material | N | Subset for alpha = 0.05 | | |
|  |  |  | 1 | 2 | 3 |
| Tukey HSD^a^ | Formslabs | 10 | .576457565167655 |  |  |
|  | Milled Chinese | 10 |  | 1.294798412795595 |  |
|  | Avadent | 10 |  | 1.597739402823873 | 1.597739402823873 |
|  | NextDent | 10 |  |  | 1.737193310657595 |
|  | Sig. |  | 1.000 | .060 | .629 |
| Means for groups in homogeneous subsets are displayed. | | | | | |
| a. Uses Harmonic Mean Sample Size = 10.000. | | | | | |

From the homogeneous subsets, we see that Formlabs is different from milled Chinese, Avadent, and Nextdent.

Milled Chinese and Avadent are not different.

Avadent and NextDent are not different.

NextDent differs from Milled Chinese and Formlabs.
