# Supplementary material for: Fracture toughness of three-dimensional printed and milled denture bases
Source: PLoS One. 2025 Aug 25;20(8):e0329556. doi: 10.1371/journal.pone.0329556 (PMC12377610; doi:10.1371/journal.pone.0329556)
Supplement: S3 File — (DOCX) [file pone.0329556.s003.docx]

**Supplemental Information 3**

**SPSS generated data**

**Explore**

| **Notes** | | |
| --- | --- | --- |
| Output Created | | 20-MAY-2025 18:56:41 |
| Comments | |  |
| Input | Data | /Users/Thamerimac/Library/Mobile Documents/com~apple~CloudDocs/All Research/Walaa Babaer/New Study Review/Walaa Statistics New Study Data.docx.sav |
|  | Active Dataset | DataSet1 |
|  | Filter | <none> |
|  | Weight | <none> |
|  | Split File | <none> |
|  | N of Rows in Working Data File | 40 |
| Missing Value Handling | Definition of Missing | User-defined missing values for dependent variables are treated as missing. |
|  | Cases Used | Statistics are based on cases with no missing values for any dependent variable or factor used. |
| Syntax | | EXAMINE VARIABLES=FToughness BY Materials /PLOT BOXPLOT HISTOGRAM NPPLOT SPREADLEVEL /COMPARE GROUPS /STATISTICS DESCRIPTIVES EXTREME /CINTERVAL 95 /MISSING LISTWISE /NOTOTAL. |
| Resources | Processor Time | 00:00:03.26 |
|  | Elapsed Time | 00:00:02.00 |

[DataSet1] /Users/Thamerimac/Library/Mobile Documents/com~apple~CloudDocs/All Research/Walaa Babaer/New Study Review/Walaa Statistics New Study Data.docx.sav

**Material**

| **Case Processing Summary** | | | | | | | |
| --- | --- | --- | --- | --- | --- | --- | --- |
|  | Material | Cases | | | | | |
|  |  | Valid | | Missing | | Total | |
|  |  | N | Percent | N | Percent | N | Percent |
| Fracture Toughness (Kmax) | FormLabs | 10 | 100.0% | 0 | 0.0% | 10 | 100.0% |
|  | Milled Chineese | 10 | 100.0% | 0 | 0.0% | 10 | 100.0% |
|  | NextDent | 10 | 100.0% | 0 | 0.0% | 10 | 100.0% |
|  | AvaDent | 10 | 100.0% | 0 | 0.0% | 10 | 100.0% |

| **Descriptives** | | | | | |
| --- | --- | --- | --- | --- | --- |
|  | Material | | | Statistic | Std. Error |
| Fracture Toughness (Kmax) | FormLabs | Mean | | .576457565167655 | .020701581689110 |
|  |  | 95% Confidence Interval for Mean | Lower Bound | .529627333868383 |  |
|  |  |  | Upper Bound | .623287796466927 |  |
|  |  | 5% Trimmed Mean | | .574772694449437 |  |
|  |  | Median | | .566925070894675 |  |
|  |  | Variance | | .004 |  |
|  |  | Std. Deviation | | .065464149305623 |  |
|  |  | Minimum | | .472073826659547 |  |
|  |  | Maximum | | .711168976603683 |  |
|  |  | Range | | .239095149944136 |  |
|  |  | Interquartile Range | | .060948158607863 |  |
|  |  | Skewness | | .486 | .687 |
|  |  | Kurtosis | | 1.442 | 1.334 |
|  | Milled Chinese | Mean | | 1.294798412795595 | .026219437888531 |
|  |  | 95% Confidence Interval for Mean | Lower Bound | 1.235485923571513 |  |
|  |  |  | Upper Bound | 1.354110902019677 |  |
|  |  | 5% Trimmed Mean | | 1.291274256543663 |  |
|  |  | Median | | 1.286115240233551 |  |
|  |  | Variance | | .007 |  |
|  |  | Std. Deviation | | .082913142697073 |  |
|  |  | Minimum | | 1.191494965827777 |  |
|  |  | Maximum | | 1.461536672298191 |  |
|  |  | Range | | .270041706470414 |  |
|  |  | Interquartile Range | | .115425088145875 |  |
|  |  | Skewness | | .675 | .687 |
|  |  | Kurtosis | | .349 | 1.334 |
|  | NextDent | Mean | | 1.737193310657595 | .126888252222483 |
|  |  | 95% Confidence Interval for Mean | Lower Bound | 1.450152142017559 |  |
|  |  |  | Upper Bound | 2.024234479297632 |  |
|  |  | 5% Trimmed Mean | | 1.749309098517874 |  |
|  |  | Median | | 1.896739190907738 |  |
|  |  | Variance | | .161 |  |
|  |  | Std. Deviation | | .401255885340970 |  |
|  |  | Minimum | | 1.115214794205744 |  |
|  |  | Maximum | | 2.141087645624427 |  |
|  |  | Range | | 1.025872851418683 |  |
|  |  | Interquartile Range | | .745077504498055 |  |
|  |  | Skewness | | -.614 | .687 |
|  |  | Kurtosis | | -1.271 | 1.334 |
|  | AvaDent | Mean | | 1.597739402823873 | .098115034467928 |
|  |  | 95% Confidence Interval for Mean | Lower Bound | 1.375787774824056 |  |
|  |  |  | Upper Bound | 1.819691030823690 |  |
|  |  | 5% Trimmed Mean | | 1.584213741277035 |  |
|  |  | Median | | 1.549948892466316 |  |
|  |  | Variance | | .096 |  |
|  |  | Std. Deviation | | .310266981624581 |  |
|  |  | Minimum | | 1.207839756020426 |  |
|  |  | Maximum | | 2.231100957470412 |  |
|  |  | Range | | 1.023261201449987 |  |
|  |  | Interquartile Range | | .442177363317662 |  |
|  |  | Skewness | | .773 | .687 |
|  |  | Kurtosis | | .559 | 1.334 |

| **Extreme Values** | | | | | |
| --- | --- | --- | --- | --- | --- |
|  | Material | | | Case Number | Value |
| Fracture Toughness (Kmax) | FormLabs | Highest | 1 | 8 | .711168976603683 |
|  |  |  | 2 | 4 | .622306064215091 |
|  |  |  | 3 | 7 | .600033350216264 |
|  |  |  | 4 | 3 | .599106937339106 |
|  |  |  | 5 | 6 | .567361283628269 |
|  |  | Lowest | 1 | 5 | .472073826659547 |
|  |  |  | 2 | 9 | .500449396906977 |
|  |  |  | 3 | 2 | .559388027841818 |
|  |  |  | 4 | 1 | .566198930104713 |
|  |  |  | 5 | 10 | .566488858161081 |
|  | Milled Chinese | Highest | 1 | 11 | 1.461536672298191 |
|  |  |  | 2 | 13 | 1.362175857840003 |
|  |  |  | 3 | 16 | 1.335939780314563 |
|  |  |  | 4 | 14 | 1.332594785029203 |
|  |  |  | 5 | 18 | 1.312941652112383 |
|  |  | Lowest | 1 | 20 | 1.191494965827777 |
|  |  |  | 2 | 17 | 1.198008618607999 |
|  |  |  | 3 | 19 | 1.236762075864065 |
|  |  |  | 4 | 12 | 1.257240891707046 |
|  |  |  | 5 | 15 | 1.259288828354718 |
|  | NextDent | Highest | 1 | 26 | 2.141087645624427 |
|  |  |  | 2 | 30 | 2.130277036092249 |
|  |  |  | 3 | 25 | 2.122590977388185 |
|  |  |  | 4 | 28 | 1.969016123262382 |
|  |  |  | 5 | 29 | 1.904443144145857 |
|  |  | Lowest | 1 | 27 | 1.115214794205744 |
|  |  |  | 2 | 23 | 1.137734228518286 |
|  |  |  | 3 | 24 | 1.460001907248766 |
|  |  |  | 4 | 21 | 1.502532012420437 |
|  |  |  | 5 | 22 | 1.889035237669618 |
|  | AvaDent | Highest | 1 | 38 | 2.231100957470412 |
|  |  |  | 2 | 35 | 1.877541261830453 |
|  |  |  | 3 | 31 | 1.752783195271781 |
|  |  |  | 4 | 34 | 1.711646417876893 |
|  |  |  | 5 | 32 | 1.611804417577586 |
|  |  | Lowest | 1 | 36 | 1.207839756020426 |
|  |  |  | 2 | 33 | 1.253379835350024 |
|  |  |  | 3 | 37 | 1.371267186341708 |
|  |  |  | 4 | 40 | 1.471937633144405 |
|  |  |  | 5 | 39 | 1.488093367355046 |

| **Tests of Normality** | | | | | | | |
| --- | --- | --- | --- | --- | --- | --- | --- |
|  | Material | Kolmogorov-Smirnov^a^ | | | Shapiro-Wilk | | |
|  |  | Statistic | df | Sig. | Statistic | df | Sig. |
| Fracture Toughness (Kmax) | FormLabs | .197 | 10 | .200^*^ | .936 | 10 | .514 |
|  | Milled Chinese | .166 | 10 | .200^*^ | .943 | 10 | .591 |
|  | NextDent | .247 | 10 | .083 | .858 | 10 | .072 |
|  | AvaDent | .138 | 10 | .200^*^ | .953 | 10 | .702 |
| *. This is a lower bound of the true significance. | | | | | | | |
| a. Lilliefors Significance Correction | | | | | | | |

| **Test of Homogeneity of Variance** | | | | | |
| --- | --- | --- | --- | --- | --- |
|  | | Levene Statistic | df1 | df2 | Sig. |
| Fracture Toughness (Kmax) | Based on Mean | 12.933 | 3 | 36 | <.001 |
|  | Based on Median | 5.992 | 3 | 36 | .002 |
|  | Based on Median and with adjusted df | 5.992 | 3 | 16.956 | .006 |
|  | Based on trimmed mean | 12.340 | 3 | 36 | <.001 |

**Fracture Toughness (Kmax)**

**Histograms**


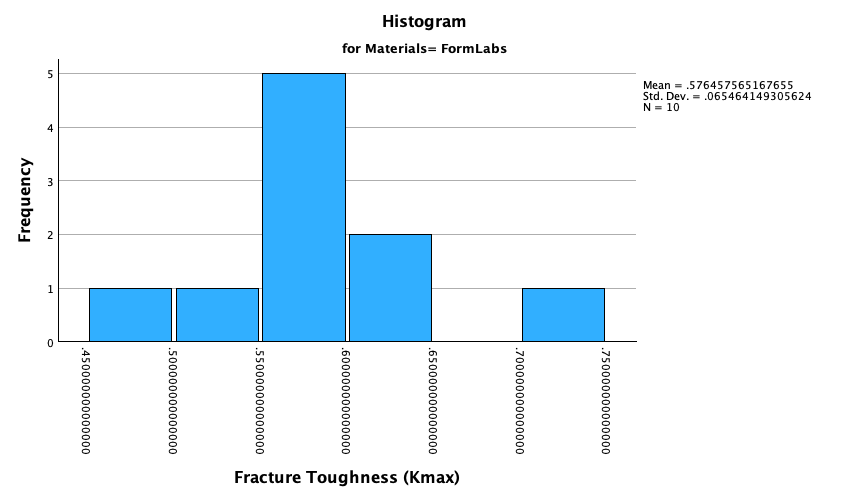


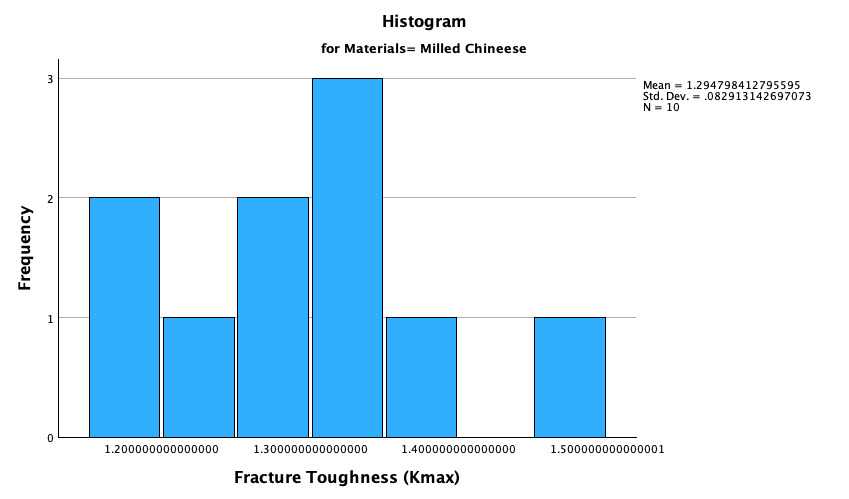


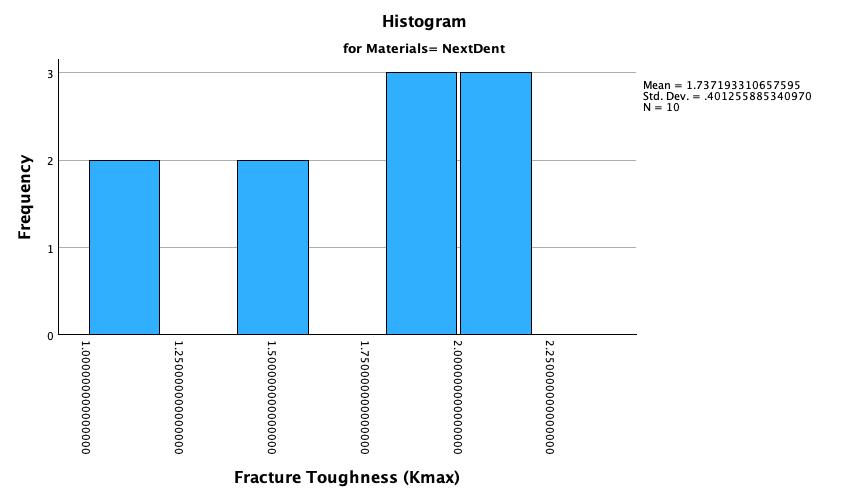


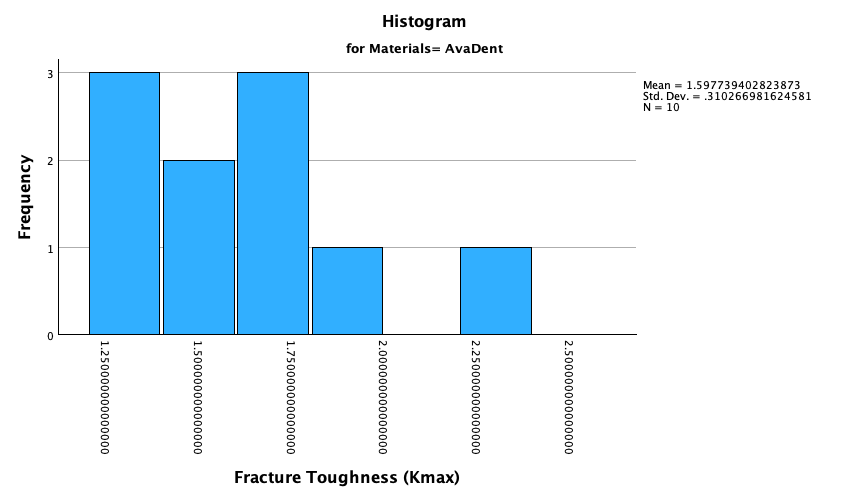


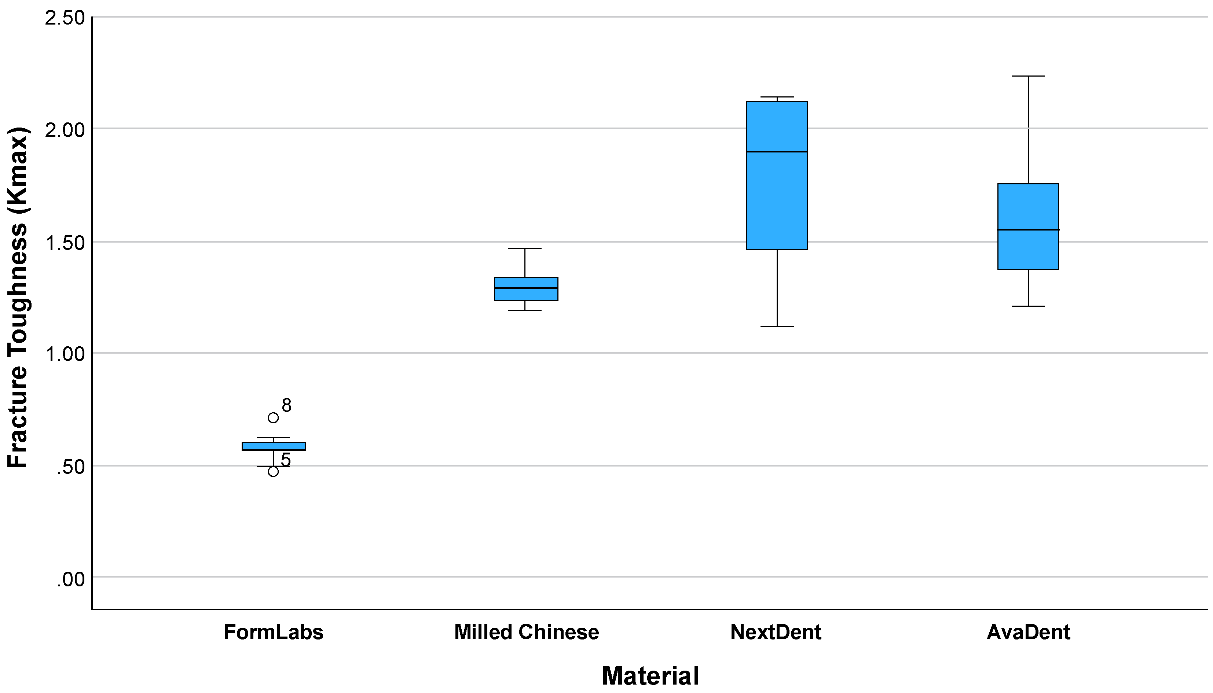


**One-way**

| **Notes** | | |
| --- | --- | --- |
| Output Created | | 20-MAY-2025 19:02:13 |
| Comments | |  |
| Input | Data | /Users/Thamerimac/Library/Mobile Documents/com~apple~CloudDocs/All Research/Walaa Babaer/New Study Review/Walaa Statistics New Study Data.docx.sav |
|  | Active Dataset | DataSet1 |
|  | Filter | <none> |
|  | Weight | <none> |
|  | Split File | <none> |
|  | N of Rows in Working Data File | 40 |
| Missing Value Handling | Definition of Missing | User-defined missing values are treated as missing. |
|  | Cases Used | Statistics for each analysis are based on cases with no missing data for any variable in the analysis. |
| Syntax | | ONEWAY FToughness BY Materials /ES=OVERALL /STATISTICS DESCRIPTIVES HOMOGENEITY /MISSING ANALYSIS /CRITERIA=CILEVEL(0.95) /POSTHOC=TUKEY BONFERRONI ALPHA(0.05). |
| Resources | Processor Time | 00:00:00.03 |
|  | Elapsed Time | 00:00:00.00 |

| **Descriptives** | | | | | | | | |
| --- | --- | --- | --- | --- | --- | --- | --- | --- |
| Fracture Toughness (Kmax) | | | | | | | | |
|  | N | Mean | Std. Deviation | Std. Error | 95% Confidence Interval for Mean | | Minimum | Maximum |
|  |  |  |  |  | Lower Bound | Upper Bound |  |  |
| FormLabs | 10 | .576457565167655 | .065464149305623 | .020701581689110 | .529627333868383 | .623287796466927 | .472073826659547 | .711168976603683 |
| Milled Chinese | 10 | 1.294798412795595 | .082913142697073 | .026219437888531 | 1.235485923571513 | 1.354110902019677 | 1.191494965827777 | 1.461536672298191 |
| NextDent | 10 | 1.737193310657595 | .401255885340970 | .126888252222483 | 1.450152142017559 | 2.024234479297632 | 1.115214794205744 | 2.141087645624427 |
| AvaDent | 10 | 1.597739402823873 | .310266981624581 | .098115034467928 | 1.375787774824056 | 1.819691030823690 | 1.207839756020426 | 2.231100957470412 |
| Total | 40 | 1.301547172861180 | .517614524909605 | .081842042435016 | 1.136006016750633 | 1.467088328971726 | .472073826659547 | 2.231100957470412 |

| **Tests of Homogeneity of Variances** | | | | | |
| --- | --- | --- | --- | --- | --- |
|  | | Levene Statistic | df1 | df2 | Sig. |
| Fracture Toughness (Kmax) | Based on Mean | 12.933 | 3 | 36 | <.001 |
|  | Based on Median | 5.992 | 3 | 36 | .002 |
|  | Based on Median and with adjusted df | 5.992 | 3 | 16.956 | .006 |
|  | Based on trimmed mean | 12.340 | 3 | 36 | <.001 |

| **ANOVA** | | | | | |
| --- | --- | --- | --- | --- | --- |
| Fracture Toughness (Kmax) | | | | | |
|  | Sum of Squares | df | Mean Square | F | Sig. |
| Between Groups | 8.033 | 3 | 2.678 | 39.902 | <.001 |
| Within Groups | 2.416 | 36 | .067 |  |  |
| Total | 10.449 | 39 |  |  |  |

| **ANOVA Effect Sizes**^a^ | | | | |
| --- | --- | --- | --- | --- |
|  | | Point Estimate | 95% Confidence Interval | |
|  |  |  | Lower | Upper |
| Fracture Toughness (Kmax) | Eta-squared | .769 | .591 | .831 |
|  | Epsilon-squared | .750 | .557 | .817 |
|  | Omega-squared Fixed-effect | .745 | .550 | .813 |
|  | Omega-squared Random-effect | .493 | .290 | .592 |
| a. Eta-squared and Epsilon-squared are estimated based on the fixed-effect model. | | | | |

**Post Hoc Tests**

| **Multiple Comparisons** | | | | | | | |
| --- | --- | --- | --- | --- | --- | --- | --- |
| Dependent Variable: Fracture Toughness (Kmax) | | | | | | | |
|  | (I) Material | (J) Material | Mean Difference (I-J) | Std. Error | Sig. | 95% Confidence Interval | |
|  |  |  |  |  |  | Lower Bound | Upper Bound |
| Tukey HSD | FormLabs | Milled Chinese | -.718340847627940^*^ | .115851635612841 | <.001 | -1.030355619605818 | -.406326075650062 |
|  |  | NextDent | -1.160735745489940^*^ | .115851635612841 | <.001 | -1.472750517467818 | -.848720973512062 |
|  |  | AvaDent | -1.021281837656218^*^ | .115851635612841 | <.001 | -1.333296609634096 | -.709267065678340 |
|  | Milled Chinese | FormLabs | .718340847627940^*^ | .115851635612841 | <.001 | .406326075650062 | 1.030355619605818 |
|  |  | NextDent | -.442394897862001^*^ | .115851635612841 | .003 | -.754409669839878 | -.130380125884123 |
|  |  | AvaDent | -.302940990028278 | .115851635612841 | .060 | -.614955762006156 | .009073781949600 |
|  | NextDent | FormLabs | 1.160735745489940^*^ | .115851635612841 | <.001 | .848720973512062 | 1.472750517467818 |
|  |  | Milled Chinese | .442394897862001^*^ | .115851635612841 | .003 | .130380125884123 | .754409669839878 |
|  |  | AvaDent | .139453907833722 | .115851635612841 | .629 | -.172560864144156 | .451468679811600 |
|  | AvaDent | FormLabs | 1.021281837656218^*^ | .115851635612841 | <.001 | .709267065678340 | 1.333296609634096 |
|  |  | Milled Chinese | .302940990028278 | .115851635612841 | .060 | -.009073781949600 | .614955762006156 |
|  |  | NextDent | -.139453907833722 | .115851635612841 | .629 | -.451468679811600 | .172560864144156 |
| Bonferroni | FormLabs | Milled Chinese | -.718340847627940^*^ | .115851635612841 | <.001 | -1.041795363739077 | -.394886331516803 |
|  |  | NextDent | -1.160735745489940^*^ | .115851635612841 | <.001 | -1.484190261601077 | -.837281229378803 |
|  |  | AvaDent | -1.021281837656218^*^ | .115851635612841 | <.001 | -1.344736353767355 | -.697827321545081 |
|  | Milled Chinese | FormLabs | .718340847627940^*^ | .115851635612841 | <.001 | .394886331516803 | 1.041795363739077 |
|  |  | NextDent | -.442394897862001^*^ | .115851635612841 | .003 | -.765849413973137 | -.118940381750864 |
|  |  | AvaDent | -.302940990028278 | .115851635612841 | .078 | -.626395506139415 | .020513526082859 |
|  | NextDent | FormLabs | 1.160735745489940^*^ | .115851635612841 | <.001 | .837281229378803 | 1.484190261601077 |
|  |  | Milled Chinese | .442394897862001^*^ | .115851635612841 | .003 | .118940381750864 | .765849413973137 |
|  |  | AvaDent | .139453907833722 | .115851635612841 | 1.000 | -.184000608277415 | .462908423944859 |
|  | AvaDent | FormLabs | 1.021281837656218^*^ | .115851635612841 | <.001 | .697827321545081 | 1.344736353767355 |
|  |  | Milled Chinese | .302940990028278 | .115851635612841 | .078 | -.020513526082859 | .626395506139415 |
|  |  | NextDent | -.139453907833722 | .115851635612841 | 1.000 | -.462908423944859 | .184000608277415 |
| *. The mean difference is significant at the 0.05 level. | | | | | | | |

**Homogeneous Subsets**

| **Fracture Toughness (Kmax)** | | | | | |
| --- | --- | --- | --- | --- | --- |
|  | Material | N | Subset for alpha = 0.05 | | |
|  |  |  | 1 | 2 | 3 |
| Tukey HSD^a^ | FormLabs | 10 | .576457565167655 |  |  |
|  | Milled Chinese | 10 |  | 1.294798412795595 |  |
|  | AvaDent | 10 |  | 1.597739402823873 | 1.597739402823873 |
|  | NextDent | 10 |  |  | 1.737193310657595 |
|  | Sig. |  | 1.000 | .060 | .629 |
| Means for groups in homogeneous subsets are displayed. | | | | | |
| a. Uses Harmonic Mean Sample Size = 10.000. | | | | | |
